# Spatial Differences in the Presence of FOXP3^+^ and GranzymeB^+^ T Cells between the Intra- and Extravascular Compartments in Renal Allograft Vasculopathy

**DOI:** 10.1371/journal.pone.0018656

**Published:** 2011-04-06

**Authors:** Onno J. de Boer, Peter Teeling, Marcel Jansen, Hanneke Ploegmakers, Chris M. van der Loos, J. Alain Kummer, Sandrine Florquin, Allard C. van der Wal

**Affiliations:** 1 Department of Pathology, Academic Medical Center (AMC), University of Amsterdam, Amsterdam, the Netherlands; 2 Department of Pathology, St. Antonius Hospital, Nieuwegein, the Netherlands; Universidade de Sao Paulo, Brazil

## Abstract

**Background:**

Allograft vasculopathy (AV) and native atherosclerosis (NA) share the presence of a T-cell mediated inflammatory response, but differ in overall plaque morphology and growth rate. We studied the distribution and frequency of regulatory- and cytotoxic T cells in the arterial intima lesions in both conditions.

**Methodology/Principal Findings:**

The study is based on vessels of 15 explanted human renal allografts with AV and 10 carotid artery plaques obtained at surgery. Distribution and frequency of cytotoxic- and regulatory T cells, as identified by the expression of Granzyme B (GrB) and FOXP3 was established in NA and AV. Furthermore, we compared the distribution of these cells in AV with the perivascular, interstitial renal tissue using immunohistochemistry. The total number of T cells was much higher in AV than in NA lesions (711±135 and 37±8 CD3/mm^2^ respectively, p<0.005, mean, ± SEM). Total numbers of FOXP3^+^ regulatory cells were also significantly increased in AV (36±10 and 0.9±0.3 FOXP3^+^/mm^2^ p<0.05), but relative numbers, expressed as a percentage of the total number of CD3^+^ T cells ((FOXP3^+^/CD3^+^) ×100), were not significantly different (4.6%±0.9 and 2.7%±0.6). GrB^+^ cells were rare in NA, but significantly increased numbers of GrB^+^ cells were found in AV lesions (85±24 and 0.2±0.1 GrB^+^/mm^2^, p<0.05). Perivascular tissues in the allografts showed a higher relative frequency of FOXP3^+^ cells than adjacent intimal lesions (14.0%±2.7 and 4.6%±0.9, respectively, p<0.05), but a lower frequency of GrB^+^ cytotoxic T cells (16.1%±2.7 and 22.6%±3.6, p<0.05).

**Conclusions:**

Similar to NA, AV is characterized by a low frequency of intimal FOXP3^+^ regulatory T cells. Moreover, significant spatial differences exist in the distribution of functional T cell subsets between the intra- and extravascular micro-environments of the graft.

## Introduction

Allograft vasculopathy (AV) is a hallmark of chronic allograft dysfunction in heart and kidney transplantation [1], [2]. AV differs from naturally occurring plaques in native arteries (native atherosclerosis, NA) by its rapid onset and progression, and distinct morphologic features like concentric rather than eccentric distribution of plaques in the affected vessels [3]. While NA is basically a lipid related chronic inflammatory process in the vessel wall, AV is generally considered to be an alloimmune process.

T cells play an important role in the pathogenesis of both NA and AV. By the secretion of inflammatory mediators and direct cell-cell interactions with other plaque cells, activated T cells are able to induce the proliferation or apoptosis of intimal cells, modulate the synthesis of extracellular matrix components and thus directly influence atherosclerotic plaque growth and stability [4]. In NA, Th1 cells, secreting high levels of interferon (IFN)-γ, are considered to be the most important T cell subset contributing to atherogenesis [4]. Th1 cells also participate in the pathogenesis of AV, but in addition, there is evidence for cytotoxic responses as well, based on the presence of Perforin and Granzyme B (GrB) positive T-cells in arteries of transplanted hearts and kidneys [5], [6].

Regulatory T cells (Treg), including the CD4^+^CD25^high^FOXPP3^+^ subset (‘natural occurring Treg’) play a central role in inducing and maintaining immunologic tolerance and the termination of immune responses [7]. In experimental transplantation it has been shown that FOXP3^+^ regulatory T cells play an important role preventing graft loss and inducing tolerance, and may prevent AV [8] but their significance in human transplantation is still a matter of debate [9].

It has been proposed that the intima of arteries represents a specialized immunological microenvironment due to the presence of specific immunocompetent cells (e.g. endothelial cells, macrophages/foam cells, T-cells, mast cells) and the typical distribution of extracellular matrix proteins [10]. For that reason it could be that one and the same initiating inflammatory factor may lead to different types of immune responses in the intima, compared to the extravascular tissue. The presence of GrB^+^ cytotoxic T cells and FOXP3^+^ regulatory T cells has been studied in heart and kidney allografts [6], [11], [12], but there are no studies that systematically compared the presence of these cells in the intima of arteries and the adjacent perivascular tissue. In this manuscript we report our quantitative immunohistochemical analysis of the *in situ* presence and distribution of FOXP3^+^ T cells and GrB^+^ cytotoxic T cells in NA and in AV.

## Methods

### Ethics Statement

The medical ethical review board granted a waiver for informed consent because only leftover tissue of normal clinical procedures, no longer required for diagnosis or clinical management was used in this study. All acquired data were analyzed anonymously. Ethics approval was obtained from the Medical Ethical Review Board of the Academic Medical Centre, University of Amsterdam.

### Specimens

This study is based on 25 paraffin embedded tissue blocks of 15 patients that underwent nephrectomy for renal failure due to chronic allograft dysfunction. Tissue blocks were retrospectively retrieved from the archives of the Pathology Department of the Academic Medical Centre. Details of the patients are summarized in Table 1. H&E and PASD stainings were prepared for histopathological grading of plaque constituents, parameters of allograft vasculopathy and histological diagnoses according to the latest Banff update '07 [13]. Small- and medium sized renal arteries (interlobar-, arcuate and interlobular arteries) were screened histomorphologically for concentric or eccentric distribution of intimal lesions and for the presence of the following plaque components: cellular intimal hyperplasia, sclerosing fibrosis, lipid core and inflammatory cells. As reference materials we sampled native atherosclerotic plaques from carotid endarterectomy specimens of 10 patients operated for symptomatic carotid artery disease (see table 1). NA specimens were handled following the same procedures as for AV lesions. All plaques were advanced, lipid/fibrolipid plaques (type IV, Va and Vb, according to the classification of the American Heart Association [14]. Plaques displaying major thrombotic events were not encountered in this series. We used carotid artery plaques since surgical materials of renal arteries with significant atherosclerosis were not available.

**Table 1:** Demographical and histopathological characteristics of the patients and nephrectomy specimens.

A: Patients / carotid enarterectomies			
ID	Sex	Age (years)	AHA classification
1	m	47	AHA Vb
2	m	65	AHA Vb
3	m	64	AHA Va
4	m	64	AHA Va
5	m	73	AHA Va
6	f	68	AHA Va
7	m	72	AHA Va
8	f	73	AHA IV
9	m	86	AHA IV
10	m	62	AHA IV

B: Patients / renal allografts				
Id	Sex	Age (years)	Graft survival (years)	Bannf classification
1	f	41	2.2	ACR IIA
2	f	41	0.7	ACR III+AHR III
3	m	30	10.3	*
4	m	44	8.7	ACR IIA+AHR II
5	m	43	0.3	ACR III**
6	m	54	0.3	ACRIIb+AHR II
7	m	15	8.5	ACRIIb+AHR II
8	m	33	17.7	ACR IIb+AHR II
9	m	35	1.0	ACR III+AHR III
10	m	29	7.8	ACR IIb+AHR II
11	f	58	14.2	ACR In+AHR II
12	m	54	1.6	ACR III+AHR III
13	f	39	2.5	ACR II+AHR II
14	m	49	0.5	ACR II+AHR II
15	f	60	0.3	ACR III

*not interpretable: no renal cortex present in the available tissue blocks to determine Banff score;

**: C4d not interpretable because of extensive haemorrhage.

### Immunohistochemistry

Immunohistochemical double stainings were performed on 5 µm thick, serial sections of all the tissue blocks. For immunohistochemical analysis, the following antibodies were used: rabbit monoclonal anti-CD3 (Pan-T, clone SP7, Thermo Scientific/LabVision, Fremont, CA, USA); rabbit monoclonal anti-CD8 (Cytotoxic T cells, clone SP16, Thermo Scientific/LabVision); mouse monoclonal anti-FOXP3 (Treg, clone 236A/E7; Abcam, Cambridge, UK); mouse monoclonal anti-Granzyme B (clone GRB-7, Monosan, Sanbio, Uden, the Netherlands [15]); mouse monoclonal anti-CD57 (NK cells, clone HNK-1, Thermo Scientific/LabVision); mouse monoclonal anti-CD68 (macrophages, PG-M1, Dako, Glostrup, Denmark); alkaline phosphatase (AP)-conjugated mouse anti-tryptase (Chemicon/Millipore, Temecula, CA, USA); rabbit polyclonal anti C4d (Biomedica Medizinproducte, Vienna, Austria) and mouse monoclonal anti- polyoma (BK) virus (clone DP02, Calbiochem/*Oncogene* Research Products, Cambridge, MA, USA). As secondary step (except for the tryptase staining, which antibody was already AP labelled) we used alkaline phosphatase conjugated anti rabbit- and anti mouse-polymers (Immunologic, Duiven the Netherlands). Immunohistochemical sequential double alkaline phosphatase staining was performed as previously described [16]. Negative control experiments were performed with matched species or mouse isotype control reagents using similar immunoglobulin concentrations.

### Quantification

Digital images covering the complete pathological intima were obtained from the histological specimens using a Leica DFC500 digital camera mounted on a Leica DM5000B microscope. T cells subsets (CD3^+^/FOXP3^−^, CD3^+^/FOXP3^+^, CD8^+^Granzyme B^−^, CD8^+^/GrB^+^) were counted in the intima of NA and AV specimens with Image Pro Plus image analysis software (Media Cybernatics, Bethesda MD, USA) using the ‘manual tag’ option. Image Pro Plus was also used for measuring the total intimal surface area of the NA and AV specimens. Only the surface area of fibrous tissue was measured (the area occupied by atheroma was not included). Furthermore, in the nephrectomy specimens 3 microscope fields of ‘inflammatory hotspots’ (areas with high density of infiltrates at scanning magnification, 20× objective) were selected in the direct peri-adventitial area of the vessels, and the numbers of T cell subsets were counted in these areas also. Of each tissue block, the total number of CD3^+^ T cells that were counted to determine the frequency of a specific T cell subset was in the range of 300–2500. The numbers of cells were expressed as mean number of cells/mm^2^ ± SEM. GrB^+^ and FOXP3^+^ cells were also expressed as a percentage of the total number of CD8^+^ or CD3^+^ cells, respectively.

### Statistical Analysis

Statistical differences between NA and AV were calculated using two tailed unpaired t-test. Differences between intra and extravascular T cells subsets in the nephrectomy specimens were analyzed using a two tailed paired t-test. Values of p<0.05 were considered statistically significant. Statistical analysis was performed using GraphPad Prism version 4.0 (GraphPad Software Inc, LaJolla, CA, USA).

## Results

### 1. Histopathological findings

All NAs lesions contained a lipid core albeit with considerable variation in volume, surrounded by collagen and other extracellular matrix components as well as smooth muscle cells (SMC) and variable degrees of inflammatory infiltrates composed of macrophages, foam cells and T lymphocytes.

**Figure 1:**
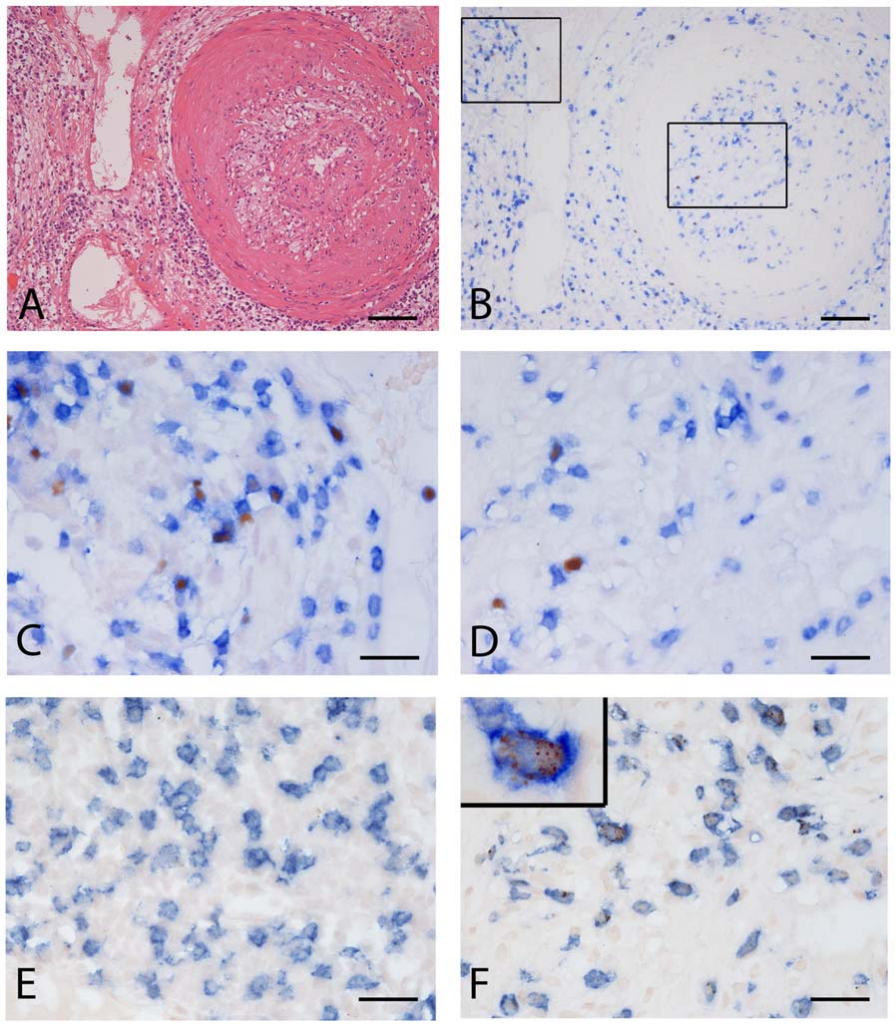
Histological examples of regulatory- and cytotoxic T cells in a nephrectomy specimen with allograft vasculopathy. All pictures are from the same specimen. A: H&E, overview. Bar = 100 µm. B: Same area as in A, doublestained with FOXP3 (red) and CD3 (blue). Bar = 100 µm. C: Detail from B, extra vascular area. Bar = 25 µm. Note the nuclear staining of FOXP3. D. Detail of B, intravascular area (intima). Bar = 25 µm. E: extra-vascular area (same region as in C), CD8 (blue)/Granzyme B(red) double staining. Bar = 25 µm. F: intima (same region as D), CD8 (blue)/Granzyme B(red) double staining,. Bar = 25 µm. Inset shows high power magnification, showing intracellular GranzymeB^+^ granules (red).

The characteristics of the nephrectomy patients are summarized in table 1, and representative histological- and immunohistochemical stainings are illustrated in figure 1. All patients showed chronic allograft vasculopathy superimposed to acute rejection (for Banff classification see table 1). None of the nephrectomies had signs of polyoma virus infections (data not shown). Interlobar arteries and small parenchymatous arteries showed concentric intimal lesions composed of SMC cells and prominent mononuclear inflammatory infiltrates, mainly macrophages and T lymphocytes. The vast majority of arteries also contained CD68^+^ foam cells and multifocal extracellular lipid deposits. Inflammatory cells were frequently found in close proximity of the endothelial lining of the vessels and in many cases “lifting up” of the endothelial layer (endothelialitis) was observed. Renal veins showed similar pathologic findings as were observed in the arteries (data not shown).

### 2. T cells and T cell-subsets in native atherosclerosis and transplantation arteriosclerosis

**Figure 2:**
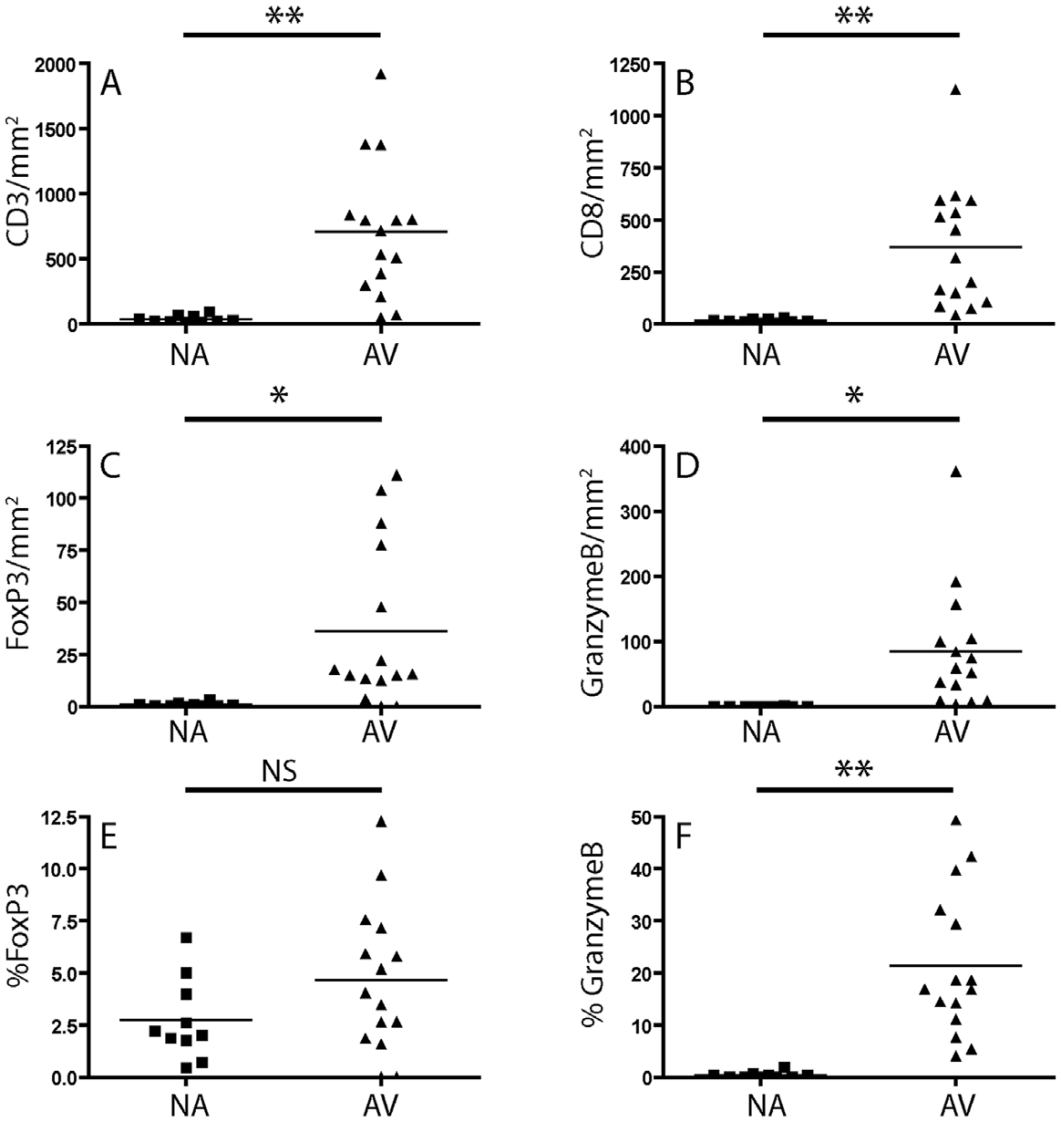
Absolute and relative numbers of CD3^+^ T cells (A), CD8^+^ T cells (B), FOXP3^+^ T cells (C and E) and Granzyme B^+^ T cells (D and F) in the intima of native atherosclerotic plaques (NA) and the intima of vessels with allograft vasculopathy (AV). *: P<0.05, ** P<0.005.

Numbers of T cells in NA and AV were assessed with the pan T cell marker CD3. The overall density of T cells in AV appeared significantly higher than in NA lesions (711±135 vs. 36.8±8.3 CD3^+^T cells/mm^2^, respectively, mean ± SEM, see figure 2A). Similarly, the numbers of CD8^+^cells/mm^2^ were also significantly higher in AV compared to NA (370±77 vs.15.3±2.7, respectively, see figure 2B).

FOXP3^+^ T cells were relatively rare in NA specimens, but significantly higher numbers were encountered in all AV specimens (0.9±0.3 vs. 36.2±10.1 FOXP3^+^/mm^2^, see figure 1B–D, and 2C). However, when the frequency of FOXP3^+^ Treg was expressed as a percentage of the total numbers of CD3^+^ cells that were present in the diseased intima, it appeared that the frequency of FOXP3^+^ T cells in AV were slightly (but not statistically significant) higher compared to NA (2.7%±0.6 and 4.6%±0.9, respectively, figure 2E).

Cytotoxic, GrB^+^ T cells were rare in native atherosclerotic plaques (0.2±0.1 cells/mm^2^). In 6/10 of the studied samples no GrB^+^ cells were encountered, while in the samples that did contain GrB^+^ cells their numbers were always very low (<5 cells per section). In sharp contrast, significantly increased numbers of GrB^+^ cells were encountered in all AV affected arteries (85±24 cells/mm^2^, see fig. 2D, and a representative histological examples in figure 1E and F ). In addition, when expressed as a percentage of the total number of CD8 positive cells present in the pathological intima, the numbers of GrB^+^ cells were significantly higher in AV when compared to NA (figure 2F)

**Figure 3:**
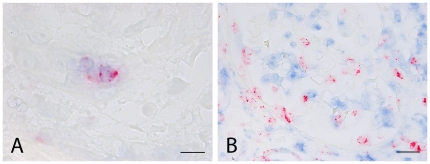
A: Immunohistochemical doublestaining of a renal allograft specimen with Granzyme B (red) and CD57 (blue), illustrating a Granzyme B positive NK cell. Bar = 10 µm. B: Doublestaining of Granzyme B (red) with CD68 (blue). Note that macrophages do not express Granzyme B. Bar = 25 µm.

Although virtually all GrB^+^ cells co-expressed CD8, sparse CD8^−^GrB^+^ cells were observed in renal allograft specimens. Most of these cells were not encountered in the affected vessels, but in the renal interstitial tissue. Immunohistochemical double staining with anti CD57 identified these cells as NK cells (see fig. 3A). GrB^+^ mast cells and/or macrophages as has been described by others, were not encountered (see figure 3B).

### 3: Intra- and extra vascular regulatory and cytotoxic T cells in chronic allograft vasculopathy

**Figure 4:**
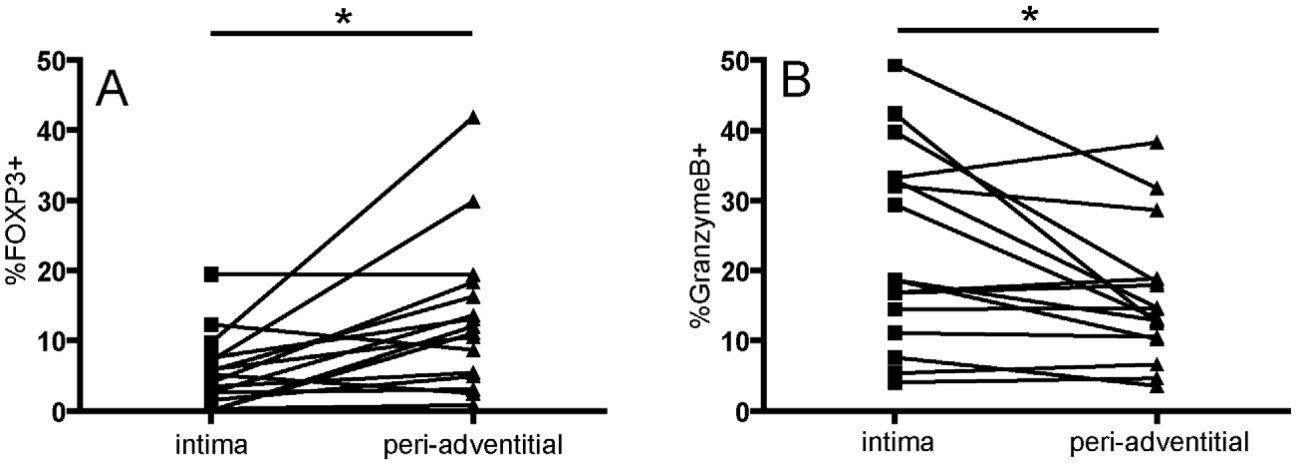
Differences between intra- and extravascular functional T cells subsets in renal allograft vasculopathy. A: numbers of FOXP3^+^ T cells, expressed as a percentage of the total number of CD3^+^ T cells. B: numbers of GrB^+^ T cells, expressed as a percentage of the total number of CD8^+^ cells. *: p<0.05, paired T test.

Finally, we also compared the intra- and extra vascular percentages of FOXP3^+^ T cells and activated cytotoxic (GrB^+^) T cells in the tissue specimens with AV. The frequency of FOXP3^+^ cells, that were found in relative low numbers in the intima of vessels with AV (4.6%±0.9 ), was significantly increased in the peri-adventitial tissue of the renal allograft specimens (14.6%±2.0, p<0.05, see figure 4A). In contrast, the frequency of GrB^+^ T cells was significantly lower in the peri-adventitial tissue (14.6%±2.0) when compared to the intima in AV(22.6±3.6, p<0.05, figure 4B).

## Discussion

In this study we investigated the presence and distribution of FOXP3^+^ regulatory- and GrB^+^ cytotoxic T cells in native and transplant arteriosclerosis, and compared the intra- and extra vascular distribution of FOXP3^+^ and GrB^+^ in renal allograft explants for chronic rejection.

Quantitative analysis of T cells revealed that the density (cells/mm^2^) of CD3^+^ and CD8^+^ T cells in the intima are more than 100-fold higher in AV compared to NA. In native atherosclerotic plaques T cells are not equally distributed throughout the intima. Local ‘hotspots’ T cells can frequently be found in ‘shoulder’ of the plaque, near the atheroma, and in the fibrous cap, and outside these areas T cells are usually sparsely present. T cells in these hotspots are considered very important because activated T cells contribute to plaque destabilization and rupture, typically on these locations [4], [17]. In AV on the other hand, T cells are more evenly distributed throughout the intima, and not as ‘hotspots’, as seen in NA. We hypothesise that due to this diffuse localisation of T cells in AV, these cells contribute to the accelerated and concentric growth pattern as seen in AV.

FOXP3^+^ T cells were more frequently encountered in AV when compared to NA, where these cells were relatively rare. However, also in AV relative numbers of FOXP3^+^ cells were low when expressed as a percentage of the total number of T cells. Hagemeijer et al recently studied FOXP3^+^ regulatory T cells in cardiac allograft explants [12], and in that study he did not observe any FOXP3^+^ Tregs in cardiac AV. Our present results, obtained in the kidney are therefore, to some extent, in variance with his results because we did encounter these cells in specimens with AV. Still, both studies clearly show that the numbers of FOXP3^+^ T cells are low in AV, which especially becomes clear when the frequency of these cells is expressed as a percentage of the total number of T cells present in the plaques.

We have previously reported on the presence of FOXP3^+^ T cells in native atherosclerosis, and observed that the numbers of Treg in native atherosclerosis, when expressed as a percentage of the total number of CD3^+^ T cells, were significantly lower when compared to normal skin and inflammatory dermatoses (psoriasis, spongiotic dermatitis, lichen planus and Leishmaniasis) [18], [19]. In normal as well as in inflammatory skin we found the frequency of Treg to be in the range of 15–25%, whereas in native atherosclerotic plaques their frequency was in the range of 1–5%, depending of the type of lesion. In the present study we found that the frequency of FOXP3^+^ cells in AV as low, and in the same range as observed in native atherosclerosis. Moreover, the frequency of FOXP3^+^ cells outside the affected arteries was significantly higher, in levels almost comparable to those previously observed in normal skin as well as chronic inflammatory skin diseases [18]. It thus appears that low frequency of FOXP3^+^ cells is also a characteristic feature for AV.

In an experimental model of atherosclerosis, Mor at al. have shown that oxidized lipids inhibit FOXP3 expression and attenuate suppressive properties of Treg [20]. Based on these findings, we hypothesized that the low frequency of Treg, as observed in native atherosclerosis was a consequence of the presence of oxidized lipids in the lesions [19]. AV lesions may also contain lipids, as macrophage foam cells are frequently encountered in this type of lesions. Therefore, the decreased frequency of Treg in AV could also be the result of lipids, that are usually present in the intima. This hypothesis is substantiated by our present observation that outside the arteries, in the interstitial renal parenchyma, the frequency of FOXP3^+^ Treg are significantly higher. Finally, further indirect support for this hypothesis is also provided by the observation that hypercholesterolemia is an independent risk factor for AV [21].

GrB staining of CTL indicates activation of an important mechanism of cell mediated cytotoxicity carried out by lytic proteins that are present in the cytoplasmic cytotoxic granules of cells [15]. Besides cytotoxic effects on target cells, Granzymes may also contribute to remodelling and weakening of the intimal extracellular matrix, by degrading extracellular matrix proteins such as proteoglycans [22]. In NA, GrB^+^ lymphocytes were very rare, implying that in this type of lesions these cells do not play a role in plaque remodelling or T-cell mediated cytotoxicity. In contrast, large numbers of GrB^+^ T cells were present in arteries of the renal explants, showing that cytotoxic T cells responses play an important role in the pathogenesis of AV.

An interesting finding of this study was that the frequency of intravascular, activated cytotoxic CD8^+^ lymphocytes was significantly higher compared to the extra-vascular compartment. We hypothesize that the increased numbers of intravascular GrB^+^ cells in AV is a consequence of the relative low frequency of FOXP3^+^ cells.

Our present findings are, to some extent, in contrast with a study by Choy et al, who also studied GrB expression in native atherosclerosis and transplant vascular disease [23]. In that study the authors showed extensive diffuse intracellular staining with GrB in macrophages, SMC (also in the media) and leukocytes in specimens with advanced native atherosclerosis and transplant arteriosclerosis. In this study, cytotoxic T cells were very rare in native atherosclerotic specimens, which is also in agreement with results already reported by others [24]. Moreover, we always observed GrB as clear intracellular granules in conjunction with CD8^+^, and occasionally also with CD57^+^ cells (figure 3). This staining pattern is exactly what one could expect, since granzymes are know to be present and stored in such cytotoxic granules. The most plausible explanation for the observed difference between our study and that of Choy is the primary antibody used. Choy and colleagues used an antibody for Santa Cruz, while we used the well characterized and frequently used GRB7 clone. Although it is known that activated mast cells are able to express GrB [25], we did not observe tryptase^+^GrB^+^ cells in any of the sections, which suggests that activated mast cells do not play an important role in AV.

Patients' immunosuppressive therapy was withdrawn (in our study group at least 2 weeks for all patients) before the surgical removal of dysfunctional allografts. Indeed, besides AV, we also observed several signs of acute rejection in these specimens. It has been shown that infiltration with GrB^+^ [25] and FOXP3^+^ [26] cells is associated with episodes of acute rejection. Therefore, it is likely that the presence of a part of these cells we observed in the allografts were the result of such a response. This could also have contributed to the observed difference in absolute numbers of functional T cell subsets between NA and AV specimens. However, this also implicates that our present finding were not, or at least to lesser extent, biased by the immunosuppressive therapy.

In conclusion, in this study we have shown that the distribution of functional T cell subsets differs between the intra- and extravascular compartment in renal allografts with chronic allograft dysfunction. The relative low numbers of FOXP3^+^ cells, and relatively high numbers of cytotoxic cells suggest that impaired T cell suppression in the arterial intima contribute to chronic allograft vasculopathy. Therapeutic enrichment of intravascular regulatory T cells, as has been proposed for the treatment of native atherosclerosis [27], may also be an interesting novel strategy to prevent chronic allograft vasculopathy.

## References

[1] Paul LC (1993). Chronic rejection of organ allografts: magnitude of the problem.. Transplantation Proceedings.

[2] Sharples LD, Caine N, Mullins P, Scott JP, Solis E (1991). Risk factor analysis for the major hazards following heart transplantation–rejection, infection, and coronary occlusive disease.. Transplantation.

[3] Rahmani M, Cruz RP, Granville DJ, McManus BM (2006). Allograft vasculopathy versus atherosclerosis.. Circulation Research.

[4] de Boer OJ, Becker AE, van der Wal AC (2003). T lymphocytes in atherogenesis - functional aspects and antigenic repertoire.. Cardiovasc Res.

[5] Colvin RB (1996). The renal allograft biopsy.. Kidney International.

[6] Fox WM, Hameed A, Hutchins GM, Reitz BA, Baumgartner WA (1993). Perforin expression localizing cytotoxic lymphocytes in the intimas of coronary arteries with transplant-related accelerated arteriosclerosis.. Human Pathology.

[7] Maggi E, Cosmi L, Liotta F, Romagnani P, Romagnani S (2005). Thymic regulatory T cells.. Autoimmun Rev.

[8] Nadig SN, Wieckiewicz J, Wu DC, Warnecke G, Zhang W (2010). In vivo prevention of transplant arteriosclerosis by ex vivo-expanded human regulatory T cells.. Nat Med.

[9] Boros P, Bromberg JS (2009). Human FOXP3+ regulatory T cells in transplantation.. Am J Transplant.

[10] Waltner-Romen M, Falkensammer G, Rabl W, Wick G (1998). A previously unrecognized site of local accumulation of mononuclear cells. The vascular-associated lymphoid tissue.. J Histochem Cytochem.

[11] Ashton-Chess J, Dugast E, Colvin RB, Giral M, Foucher Y (2009). Regulatory, effector, and cytotoxic T cell profiles in long-term kidney transplant patients.. J Am Soc Nephrol.

[12] Hagemeijer MC, van Oosterhout MF, van Wichen DF, van Kuik J, Siera-de Koning E (2008). T cells in cardiac allograft vasculopathy are skewed to memory Th-1 cells in the presence of a distinct Th-2 population.. Am J Transplant.

[13] Solez K, Colvin RB, Racusen LC, Haas M, Sis B (2008). Banff 07 Classification of Renal Allograft Pathology: Updates and Future Directions.. American Journal of Transplantation.

[14] Stary HC, Chandler AB, Dinsmore RE, Fuster V, Glagov S (1995). A Definition of Advanced Types of Atherosclerotic Lesions and a Histological Classification of Atherosclerosis : A Report From the Committee on Vascular Lesions of the Council on Arteriosclerosis, American Heart Association.. Arterioscler Thromb Vasc Biol.

[15] Kummer JA, Kamp AM, van Katwijk M, Brakenhoff JP, Radosevic K (1993). Production and characterization of monoclonal antibodies raised against recombinant human granzymes A and B and showing cross reactions with the natural proteins.. Journal of Immunological Methods.

[16] de Boer OJ, van der Meer JJ, Teeling P, van der Loos CM, Idu MM (2010). Differential expression of interleukin-17 family cytokines in intact and complicated human atherosclerotic plaques.. Journal of Pathology.

[17] Hosono M, de Boer OJ, van der Wal AC, van der Loos CM, Teeling P (2003). Increased expression of T cell activation markers (CD25, CD26, CD40L and CD69) in atherectomy specimens of patients with unstable angina and acute myocardial infarction.. Atherosclerosis.

[18] de Boer OJ, van der Loos CM, Teeling P, van der Wal AC, Teunissen MB (2007). Immunohistochemical Analysis of Regulatory T Cell Markers FOXP3 and GITR on CD4+CD25+ T Cells in Normal Skin and Inflammatory Dermatoses.. J Histochem Cytochem.

[19] de Boer OJ, van der Meer JJ, Teeling P, van der Loos CM, van der Wal AC (2007). Low numbers of FOXP3 positive regulatory T cells are present in all developmental stages of human atherosclerotic lesions.. PLoS ONE.

[20] Mor A, Planer D, Luboshits G, Afek A, Metzger S (2007). Role of naturally occurring CD4+ CD25+ regulatory T cells in experimental atherosclerosis.. Arterioscler Thromb Vasc Biol.

[21] Kobashigawa JA, Kasiske BL (1997). Hyperlipidemia in solid organ transplantation.. Transplantation.

[22] Ronday HK, van der Laan WH, Tak PP, de Roos JA, Bank RA (2001). Human granzyme B mediates cartilage proteoglycan degradation and is expressed at the invasive front of the synovium in rheumatoid arthritis.. Rheumatology (Oxford).

[23] Choy JC, McDonald PC, Suarez AC, Hung VH, Wilson JE (2003). Granzyme B in atherosclerosis and transplant vascular disease: association with cell death and atherosclerotic disease severity.. Mod Pathol.

[24] Dong C, Wilson JE, Winters GL, McManus BM (1996). Human transplant coronary artery disease: pathological evidence for Fas-mediated apoptotic cytotoxicity in allograft arteriopathy.. Laboratory Investigation.

[25] Strik MC, de Koning PJ, Kleijmeer MJ, Bladergroen BA, Wolbink AM (2007). Human mast cells produce and release the cytotoxic lymphocyte associated protease granzyme B upon activation.. Mol Immunol.

[26] Yapici U, Bemelman FJ, Scheepstra CG, Roelofs JJ, Claessen N (2009). Intragraft FOXP3 protein or mRNA during acute renal allograft rejection correlates with inflammation, fibrosis, and poor renal outcome.. Transplantation.

[27] Klingenberg R, Hansson GK (2009). Treating inflammation in atherosclerotic cardiovascular disease: emerging therapies.. European Heart Journal.

